# The Role of a Single Angiogenesis Inhibitor in the Treatment of Recurrent Glioblastoma Multiforme: A Meta-Analysis and Systematic Review

**DOI:** 10.1371/journal.pone.0152170

**Published:** 2016-03-23

**Authors:** Yawei Wang, Dan Xing, Meng Zhao, Jie Wang, Yang Yang

**Affiliations:** 1 Department of Electromyography, Tianjin Hospital, Tianjin, China; 2 Arthritis Clinic & Research Center, Peking University People's Hospital, Peking University, Beijing, China; 3 Clinical laboratory, Tianjin Medical University Cancer Institute and Hospital, National Clinical Research Center for Cancer, Key Laboratory of Cancer Prevention and Therapy, Tianjin, China; 4 Department of Orthopedics, Tianjin Hospital, Tianjin, China; University of California-San Francisco, UNITED STATES

## Abstract

**Background:**

Currently, the standard treatment for newly diagnosed glioblastoma multiforme (GBM) is maximal safe surgical resection followed by radiation therapy with concurrent and adjuvant temozolomide. However, disease recurs in almost all patients, and the optimal salvage treatment for recurrent GBM remains unclear. We conducted a systematic review and meta-analysis of published clinical trials to assess the efficacy and toxicities of angiogenesis inhibitors alone as salvage treatment in these patients.

**Methods:**

Trials published between 1994 and 2015 were identified by an electronic search of public databases (MEDLINE, EMBASE, Cochrane library). Demographic data, treatment regimens, objective response rate (ORR), median progression-free survival (PFS), median overall survival (OS), 6-months PFS rate, 1-year OS and grade 3/4 toxicities were extracted. We also compared the main outcomes of interest between bevacizumab and other angiogenesis inhibitors. All analyses were performed using Comprehensive Meta Analysis software (Version 2.0).

**Results:**

A total of 842 patients were included for analysis: 343 patients were treated with bevacizumab, 386 with other angiogenesis inhibitors and 81 with thalidomide. The pooled ORR, 6-months PFS, and 1-year OS for recurrent GBM patients receiving angiogenesis inhibitors was 20.1%, 19.5% and 29.3%, respectively. The use of single agent bevacizumab in recurrent GBM significantly improved ORR and 6-months PFS when compared to other angiogenesis inhibitors [relative risk (RR) 2.93, 95% CI 1.38–6.21; *p* = 0.025; and RR 2.36 95% CI 1.46–3.82; *p*<0.001, respectively], while no significant difference in 1-year OS was found between the two groups (*p* = 0.07). when compared to thalidomide, bevacizumab treatment in recurrent GBM significantly improved ORR (RR 6.8, 95%CI: 2.64–17.6, p<0.001), but not for 6-months PFS (*p* = 0.07) and 1-year OS (*p* = 0.31). As for grade 3/4 toxicities, the common toxicity was hypertension with pooled incidence of 12.1%, while high-grade thromboembolic events (2.2%), hemorrhage (5.1%) and GI perforation (2.8%) associated with angiogenesis inhibitors were relatively low.

**Conclusions:**

In comparison with other angiogenesis inhibitors and thalidomide, the use of single agent bevacizumab as salvage treatment for recurrent GBM patients improve ORR and 6-months PFS, but not for 1-year OS.

## Introduction

Glioblastoma multiforme (GBM) is the most common malignant primary brain tumor in adults, with an average incidence rate of more than 3/100,000 individuals each year [1, 2]. The current standard of care is maximal safe surgical resection followed by adjuvant concomitant chemoradiotherapy and subsequent consolidation chemotherapy, generally with temozolomide [3, 4]. Despite this multimodality treatment approach, nearly all patients experience disease progression. And the prognosis of recurrent GBM remains dismal, with a median survival of only 14 to 16 months, with 5-year overall survival rate less than 10% [5–7]. For patients with recurrent GBM, salvage chemotherapeutic or biological agents are the most common approach for second-line treatment as most of these patients will not be candidates for new surgery or re-irradiation.

Previous research has found that GBM is a highly vascularized tumor in which micro-vascular proliferation is typically observed [8–10], and vascular endothelial growth factor (VEGF) has been identified as a prominent mediator of tumor angiogenesis [11, 12]. Thus, angiogenesis inhibitors targeting the VEGF signal pathway obtain a focus of significant scientific interest. Bevacizumab, a humanized antibody to VEGF, received accelerated US Food and Drug Administration (FDA) approval in May 2009 for use as a single agent in patients with GBM who have progressive disease following front-line therapy consisting of surgical resection, radiotherapy, and temozolomide[4, 13, 14]. In an attempt to improve treatment outcomes, several novel angiogenesis inhibitors have been investigated in prospective clinical trials. However, to our best knowledge, no systematic review focusing on the efficacy and toxicities associated with angiogenesis inhibitors alone in recurrent GBM has been performed, and whether bevacizumab is more efficient than other angiogenesis inhibitors and thalidomide remains unknown. Therefore, we perform a systematic review and meta-analysis of published data to compared treatment outcomes with single agent bevacizumab versus other angiogenesis inhibitors and thalidomide for recurrent GBM patients.

## Method and Materials

### Search strategy and selection of trials

We Performed this meta-analysis adheres to the Preferred Reporting Items for Systematic Reviews and Meta-Analyses (PRISMA) statements[15] (S1 table). To identify studies for inclusion in our systematic review and meta-analysis, we did a broad search of four databases, including Embase, Medline, the Cochrane Central Register of Controlled Trials, and the Cochrane Database of Systematic Reviews, from the date of inception of every database to July 2015. The complete search strategy employed has been provided (S1 Text). No language restrictions were applied.

To be eligible for inclusion in our systematic review and meta-analysis, study populations (referred to hereafter as cohorts) had to meet all the following criteria: 1) patients with recurrent glioblastoma refractory to previous treatments; 2) treatment with angiogenesis inhibitors alone; 3) reported outcomes of interest (ie, objective response rate, 6-months PFS and 1-year OS; and 4) from an original prospective study (ie, randomized controlled trial and non-randomized clinical trial).

### Data extraction

Two investigators screened the titles and abstracts of potentially relevant studies. We retrieved the full text of relevant studies for further review by the same two reviewers. A third senior investigator resolved any discrepancies between reviewers. The same pair of reviewers extracted study details independently, using a standardized pilot-tested form. A third investigator reviewed all data entries. We extracted the following data: author, study design, study period, median age, interventions (angiogenesis inhibitors regimen and dose), sample size and outcomes of interest. We defined outcomes of interest as objective response rate (ORR), 6-months progression-free survival (PFS), and 1-year overall survival (OS). To assess quality, since we included non-comparative (uncontrolled) studies in our systematic review and meta-analysis, we used the Newcastle-Ottawa quality assessment scale[16]. We selected items that focused on representativeness of study patients, demonstration that the outcome of interest was not present at the start of the study, adequate assessment of outcome, sufficient length of follow-up to allow outcomes to arise, and adequacy of follow-up.

### Statistical analysis

We prespecified the analysis plan in the protocol. We analyzed all patients who started angiogenesis inhibitors alone, regardless of their adherence to treatment. We calculated event rates of outcome (the proportion of patients who developed outcomes of interest) from the included cohorts. We pooled log-transformed event rates with DerSimonian and Laird random-effect models and assessed heterogeneity using *X*^2^-based Q statistic test[17]. We used the test of interaction proposed by Altman and Bland[18] to compare log-transformed rates of outcomes between bevacizumab and other angiogenesis inhibitors. A statistical test with a *p*-value less than 0.05 was considered significant. To measure overall heterogeneity across the included cohorts, we calculated the *I*^2^ statistic, with *I*^2^ greater than 50% indicating high heterogeneity. We did all statistical analyses with comprehensive meta-analysis software version 2.0(Biostat, Englewood, NJ, USA).

## Results

### Search results

**Fig 1:**
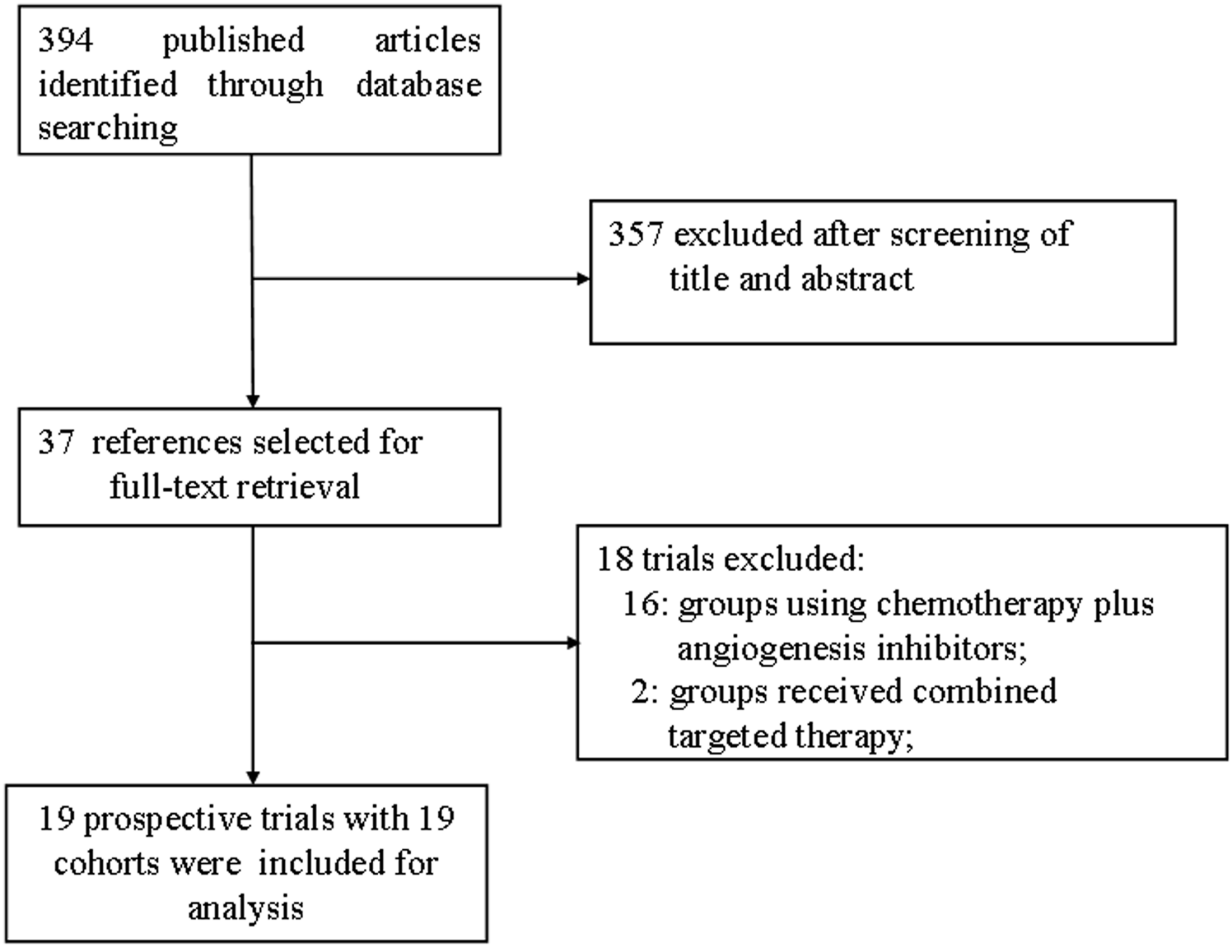
Selection process for clinical trials included in the meta-analysis.

A total of 394 studies were identified from the database search, of which 37 reports were retrieved for full-text evaluation. 19 cohorts from 19 trials met the inclusion criteria and were included in this systematic review [4, 19–36] (Fig 1). We did not find randomized controlled trials or controlled studies that directly compared single agent bevacizumab with other angiogenesis inhibitors in recurrent GBM patients. Table 1 showed the characteristics of the included studies. Overall, 842 recurrent GBM patients were included, with a median age of 54 years. The median PFS in the bevacizumab group was 3 months (95% CI, 1.89–3.95 months), and in the other angiogenesis inhibitors group was 2.45 months (95% CI, 1.32–3.76 months). The median OS was also higher in single agent bevacizumab than other angiogenesis inhibitors cohorts. The median OS in the bevacizumab group was 8.5 months (95% CI, 6.85–11.25 months), and in the other angiogenesis inhibitors group was 7.2 months (95% CI, 6.25–8.38 months).

**Table 1:** Baseline characteristics of 19 cohort groups for meta-analysis.

Author	Year	Patients, n	Treatment regimens	Median age, y	Median OS, months	Median PFS, months	1-year OS
**Norden A.D. et al**	2015	36	Nintedanib 200mg bid po	54	6.9	1	NR
**Taal W. et al**	2014	50	Bevacizumab 5mg/kg/w	58	8	3	26%
**Hutterer M. et al**	2014	40	Sunitinib 37.5mg qd po	58	9.2	2.2	27.5
**Hassler M.R. et al**	2014	30	Sorafenib 400mg bid po	46.5	6	4.1	NR
**Muhic A. et al**	2013	25	Nintedanib 200mg bid po	55	6	1	NR
**Batchelor T.T. et al**	2013	131	Cediranib 30 mg qd po	54	8	3.1	31.60%
**Pan E. et al**	2012	16	Sunitinib 37.5mg qd po	57.8	12.6	1.4	47.7
**Nagane M.et al**	2012	29	Bevacizumab 5mg/kg/w	57	10.5	3.3	34.5
**Kreisl T.N. et al**	2012	32	Vandetanib 300mg qd po	47	6.3	1.3	NR
**Kreisl T.N. et al**	2011	31	Bevacizumab 5mg/kg/w	44	12	2.93	NR
**de Groot J.F. et al**	2011	42	Aflibercept 4mg/kg/q.2.w	55	NR	5.4	35%
**Raizer J.J. et al**	2010	50	Bevacizumab 5mg/kg/w	52	6.5	2.8	NR
**Iwamoto F.M. et al**	2010	35	Pazopanib 800mg qd po	53	8.2	2.8	32%
**Chamberlain M.C. et al**	2010	50	Bevacizumab 5mg/kg/w	64	8.5	1	22%
**Batchelor T.T. et al**	2010	31	Cediranib 45 mg qd po	53	7.6	3.9	NR
**Kreisl T.N. et al**	2009	48	Bevacizumab 5mg/kg/w	53	7.2	3.7	NR
**Friedman H.S. et al**	2009	85	Bevacizumab 5mg/kg/w	54	9.2	4.2	23%
**Marx G.M. et al**	2001	42	Thalidomide 100mg qd po	55	7.2	2.6	35%
**Fine H.A. et al**	2000	39	Thalidomide 800mg qd po	49	6.5	2.3	20.50%

Abbreviations: OS, overall survival; PFS, progression free survival; ORR, objective response rate; NR, not reported;

**Fig 2:**
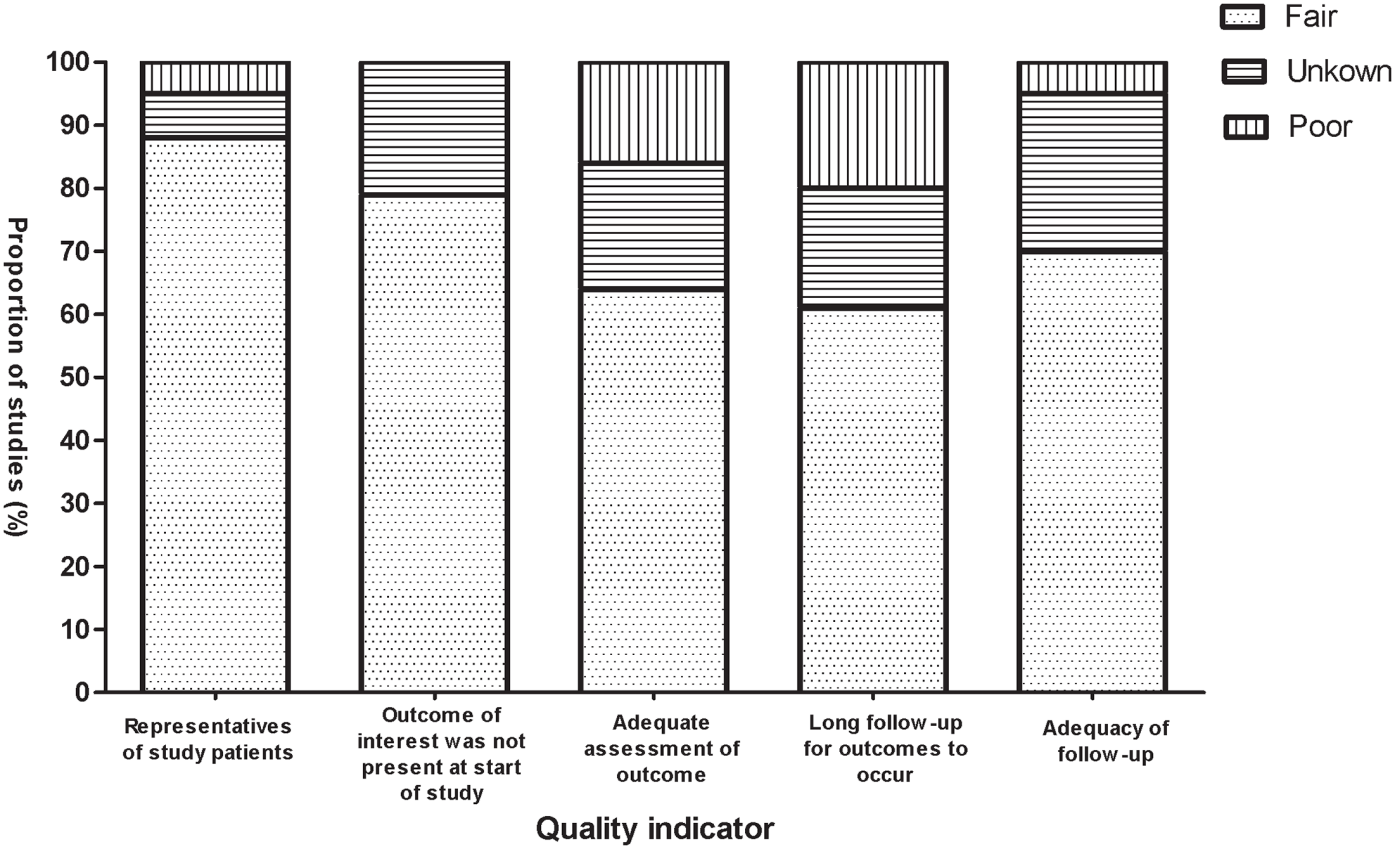
Selected methodological quality indicator.

Methodological quality of the included studies was fair; most studies provided adequate outcome ascertainment, enrolled a representative sample of patients, and had an acceptable length of follow-up (Fig 2). However, comparative evidence was at high risk of bias because we compared data across studies not within them, and selection bias was likely to be present.

### Pooled incidence of primary outcomes

**Fig 3:**
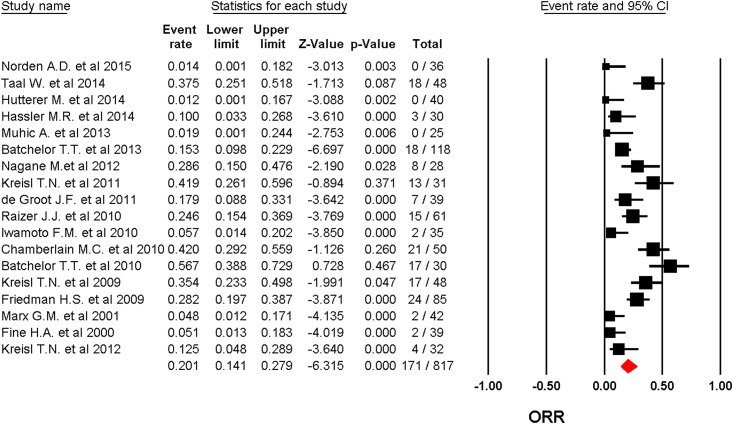
random-effects Model of pooled ORR (95% confidence interval) associated with angiogenesis inhibitors in recurrent GBM.

**Fig 4:**
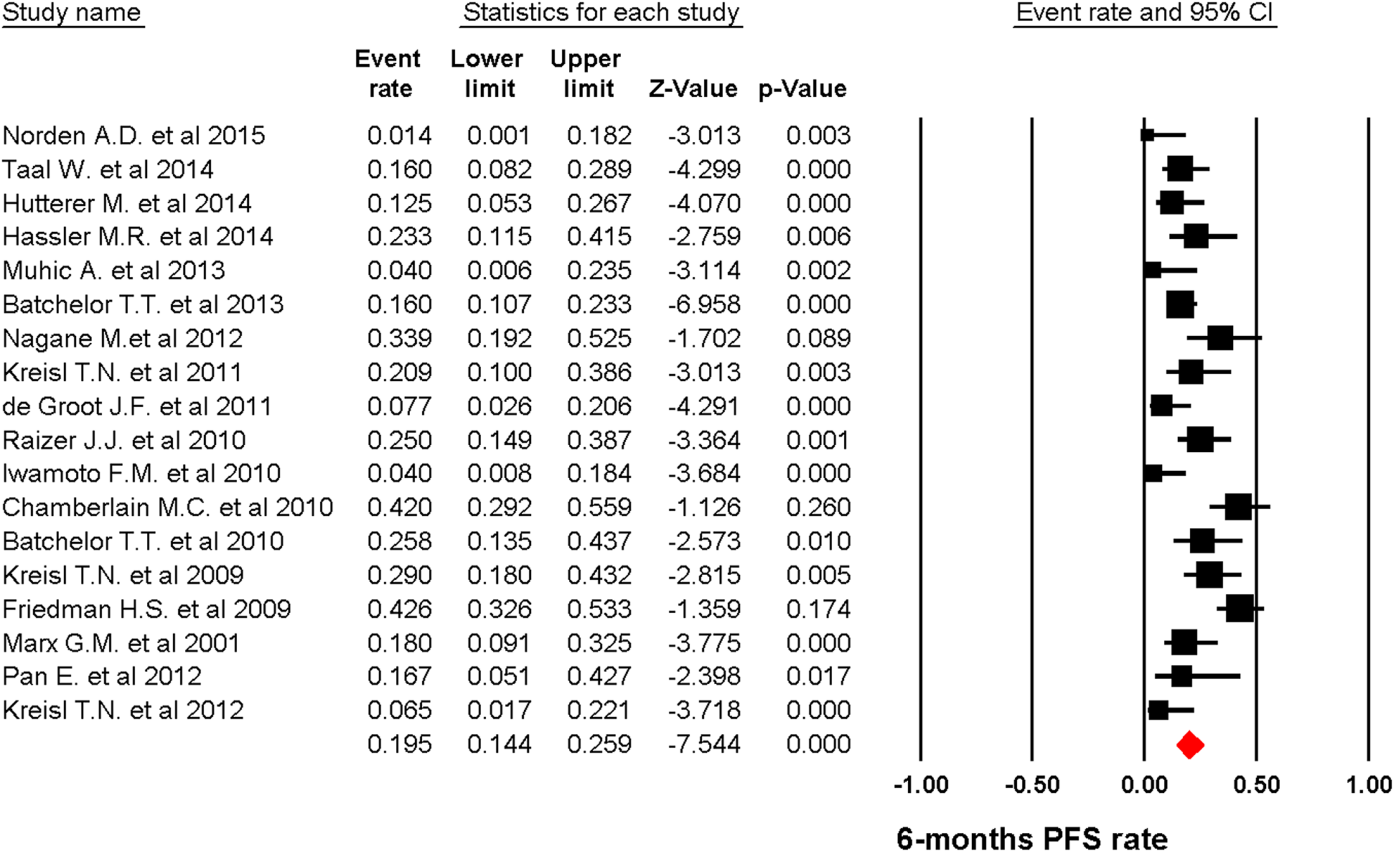
random-effects Model of pooled PFS (95% confidence interval) associated with angiogenesis inhibitors in recurrent GBM.

**Fig 5:**
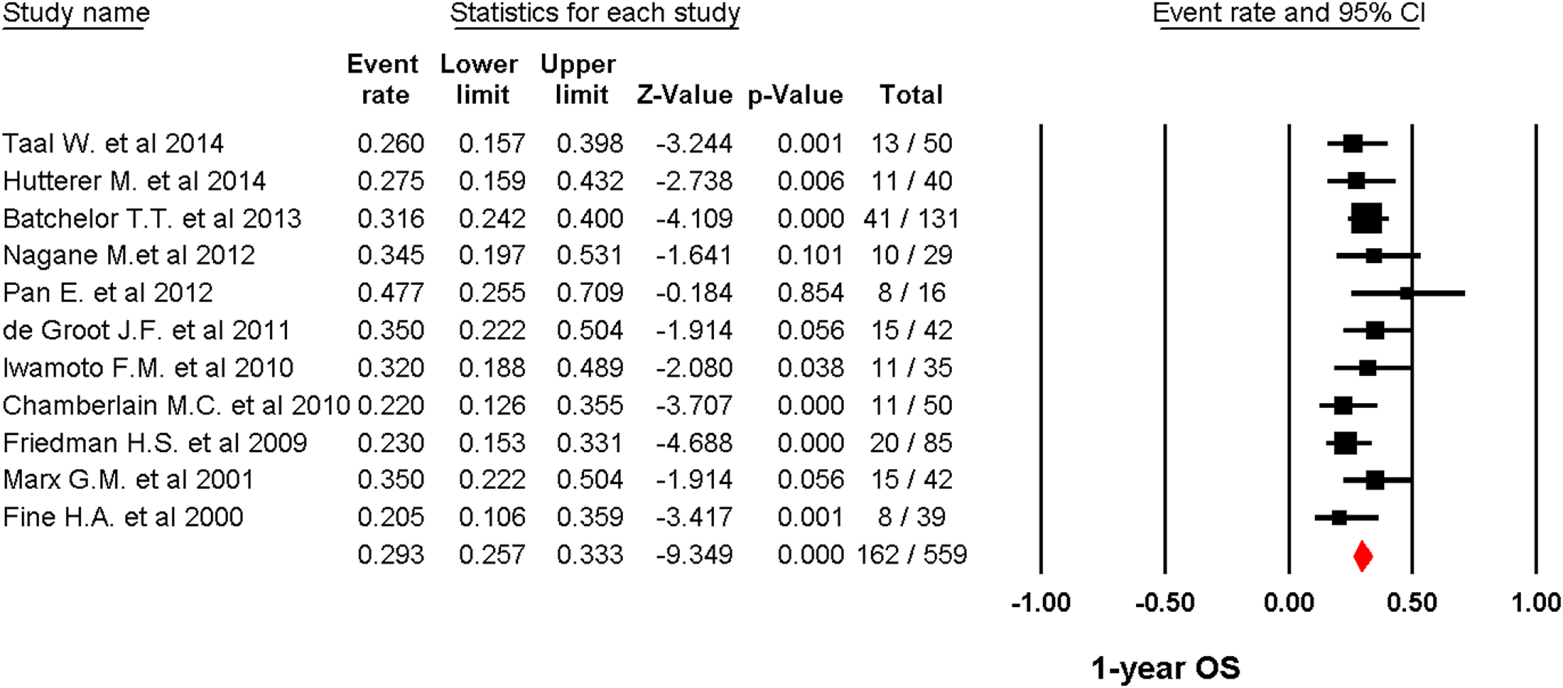
Fixed-effects Model of pooled 1-OS (95% confidence interval) associated with angiogenesis inhibitors in recurrent GBM.

A total of 817 patients were included for ORR analysis. The pooled event rate of ORR for recurrent GBM patients receiving angiogenesis inhibitors was 20.1% (95%CI: 14.1–27.9%, Fig 3) using a random effect model (*I*2 = 78.1%). A total of 803 patients form eighteen trials reported 6-months PFS rate. The pooled 6-months PFS for angiogenesis inhibitors was 19.5% (95%CI: 14.4–25.9%, Fig 4) using a random effect model (*I*2 = 71.8%). Eleven trials with a total of 559 patients were included for OS analysis yielding a pooled 1-year OS of 29.3% (95%CI: 25.7–33.3%, Fig 5) using a fixed effect model (*I*2 = 0%).

#### Efficacy comparison between bevacizumab and other angiogenesis inhibitors

The pooled event rate of ORR and 6-months PFS for recurrent GBM patients receiving single agent bevacizumab was significantly higher than that for other angiogenesis inhibitors (RR 2.93, 95% CI 1.38–6.21; *p* = 0.025; and RR 2.36 95% CI 1.46–3.82; *p*<0.001, Table 2), while there was no significantly difference in 1-year OS between the two groups (RR 0.60, 95%CI: 0.62–1.08; p = 0.07, Table 2).

**Table 2:** Comparison of primary outcomes for bevacizumab versus other angiogenesis inhibitors.

Groups	Cohorts (n)	Patients (n)	Events (95%)	*I*^2^	Relative risk (95%)	*p*
**ORR**						
**Other angiogenesis inhibitors**	11	385	11.4 (5.3–23.1)	79.9	1	
**Bevacizumab**	7	351	33.4(28.6–38.5)	7.7	2.93(1.38–6.21)	0.025
**6-months PFS**						
**Other angiogenesis inhibitors**	10	418	12.8(8.5–18.9)	43.7	1	
**Bevacizumab**	7	343	30.2(22.8–38.9)	60.3	2.36(1.46–3.82)	<0.001
**1-year OS**						
**Other angiogenesis inhibitors**	5	277	31.9(26.7–37.7)	0	1	
**Bevacizumab**	4	201	25.7(20.1–32.3)	32.4	0.60 (0.62–1.08)	0.07

*I*^2^≥50% suggests high heterogeneity across studies.

Abbreviation: ORR, objective response rate; PFS, progression-free survival; OS, overall survival.

#### Efficacy comparison between bevacizumab and thalidomide

The pooled event rate of ORR for recurrent GBM patients receiving single agent bevacizumab was significantly higher than that for thalidomide (RR 6.8, 95% CI 2.64–17.6; *p*<0.001, Table 3), and there was a tendency to improve 6-months PFS (RR 1.68, 95%CI: 0.84–3.34, p = 0.07), while there was no significant difference in 1-year OS between the two groups (RR 0.89, 95%CI: 0.59–1.37; p = 0.31, Table 3).

**Table 3:** Comparison of primary outcomes for bevacizumab versus thalidomide.

Groups	Cohorts (n)	Patients (n)	Events (95%)	*I*^2^	Relative risk (95%)	*p*
**ORR**						
**Thalidomide**	2	81	4.9 (1.9–12.4)	0	1	
**Bevacizumab**	7	351	33.4(28.6–38.5)	7.7	6.8 (2.64–17.6)	<0.001
**6-months PFS**						
**Thalidomide**	1	42	18.0 (9.1–32.5)	0	1	
**Bevacizumab**	7	343	30.2(22.8–38.9)	60.3	1.68 (0.84–3.34)	0.07
**1-year OS**						
**Thalidomide**	2	81	28.6(19.7–39.6)	51.7	1	
**Bevacizumab**	4	201	25.7(20.1–32.3)	32.4	0.89 (0.59–1.37)	0.31

*I*^2^≥50% suggests high heterogeneity across studies.

Abbreviation: ORR, objective response rate; PFS, progression-free survival; OS, overall survival.

### Toxicity

Table 4 showed the overall occurrence of high-grade (≥grade 3) anti-VEGF toxicities with angiogenesis inhibitors. The common high-grade toxicities associated with angiogenesis inhibitors were hypertension with pooled incidence of 12.1% (95%CI: 9.6–15.1%), while high-grade thromboembolic events (2.2%, 95%CI: 1.2–3.9%), hemorrhage (5.1%, 95%CI: 3.6–7.2%) and GI perforation (2.8%, 95%CI: 1.2–6.4%) associated with angiogenesis inhibitors were relatively low in recurrent GBM patients (table 4).

**Table 4:** Anti-VEGF related grade 3/4 toxic event rates for single angiogenesis inhibitors.

Toxicities	Included study	Events	Total	Events rate (95%CI)	*I*^*2*^
**Hypertension**	14	66	670	12.1 (9.6–15.1)	47.0
**Thromboembolic events**	13	7	616	2.2 (1.2–3.9)	0
**Hemorrhage**	14	29	690	5.1 (3.6–7.2)	0
**GI perforation**	5	5	251	2.8 (1.2–6.4)	0

### Publication bias

The Begg’s and Egger’s test did not reveal any evidence of obvious asymmetry for 1-year OS (Begg’s test: *p* = 0.58 and Egger’s test: *p* = 0.73, respectively), while an obvious publication was observed for ORR (Begg’s test: *p* = 0.034 and Egger’s test: *p* = 0.003, respectively) and 6-month PFS (Begg’s test: *p* = 0.002 and Egger’s test: *p* = 0.001, respectively).

## Discussion

GBM is the most common and aggressive primary brain tumor. After tumor progression on first-line therapy with concomitant chemoradiotherapy followed by consolidation temozolomide, there are few effective treatment options for these patients with. Due to the aggressive and rapid fatal disease course of recurrent GBM, the development of novel drugs for these patients is badly needed. In recent years, angiogenesis inhibitors targeted VEGF signal pathways have become a focus for the management of recurrent GBM, and bevacizumab has been approved for use as single agent in these patients[37, 38]. However, to the best of our knowledge, there is lack of meta-analysis to assess the efficacy and toxicities associated with angiogenesis inhibitors in recurrent GBM.

A total 842 recurrent GBM patients from 19 trials were included for analysis: 343 patients were treated with bevacizumab alone and 467 with other angiogenesis inhibitors. Based on our pooled results, we find that the use of single agent bevacizumab in recurrent GBM significantly improves ORR and 6-months PFS when compared to other angiogenesis inhibitors and thalidomide, while no significant difference in 1-year OS was found between the two groups. According to our results, single agent bevacizumab seems superior to other angiogenesis inhibitors and thalidomide as salvage treatment for these patients in terms of ORR and 6-months PFS, but the overall survival, which does reflect a direct clinical benefit for the patient (as opposed to ORR), of bevacizumab is not superior to other angiogenesis inhibitors and thalidomide. As a result clinicians should be cautious when interpreting these results due to the limitations of our studies, and randomized controlled trials directly comparing the efficacy between bevacizumab and other angiogenesis inhibitors in this setting are needed. Recently, several trials have been conducted to investigate whether bevacizumab alone should be preferred over combination therapy, but the results are controversial. In a phase II trial conducted by Taal W. et al [39], the authors showed that the combination of bevacizumab with lomustine was more effective than bevacizumab. Unfortunately, another recently published results of the EORTC 26101 study[40], a phase III study evaluating the efficacy of bevacizumab combined with lomustine in recurrent GBM showed no evidence of improved overall survival. Therefore, further studies are still needed to investigate whether bevacizumab-based combination therapy is superior to bevacizumab in recurrent GBM.

Safety of systematic treatments is of particular importance in palliative setting in recurrent GBM patients, given the potential negative impact on benefit ratio and quality of life. Finding of our study indicates that the common grade 3/4 toxicities associated with angiogenesis inhibitors were hypertension with pooled incidence of 12.1%, while high-grade thromboembolic events, hemorrhage and GI perforation associated with AAs were relatively low.

Several limitations need to be mentioned in this analysis. First, using formal meta-analytic methods to pool the observational studies remains controversial, because the trial designs and populations of these studies are diverse, which could influence the pooled estimates. However, there is no published data regarding comparison of single agent bevacizumab versus other angiogenesis inhibitors and thalidomide, and a meta-analysis of observational studies could comprehensively assess the overall efficacy and toxicities of angiogenesis inhibitors in recurrent GBM[41]. Second, the inclusion criteria for clinical trials likely favor young, fit, and responder patients, a highly selected group of subjects with good prognostic indicators, all of these might cause potential selection bias. Thirdly, we use the DerSimonian and Laird model to pool the overall outcomes due to significant heterogeneity among included studies. However, this model has its limitations, especially when the number of studies is smaller than 20. Therefore, the variance between studies might be negatively biased and the method also does not take uncertainty into account. Finally, this meta-analysis only considers published literature, and lack of individual patient data prevents us from adjusting the treatment effect according to previous treatment and patient variables.

## Conclusions

In comparison with other angiogenesis inhibitors and thalidomide, the use of single agent bevacizumab as salvage treatment for recurrent GBM patients improve ORR and 6-months PFS, but not for 1-year OS.

## Supporting Information

S1 Table PRISMA checklist. (DOC)

S1 Text Search strategy used to identify trials; (DOCX)
