# Supplementary material for: The Role of a Single Angiogenesis Inhibitor in the Treatment of Recurrent Glioblastoma Multiforme: A Meta-Analysis and Systematic Review
Source: PLoS One. 2016 Mar 23;11(3):e0152170. doi: 10.1371/journal.pone.0152170 (PMC4805294; doi:10.1371/journal.pone.0152170)
Supplement: S1 Text — (DOCX) [file pone.0152170.s002.docx]

S1 Text. Search strategy used to identify trials;

Search strategy used the following limits alone, or in combination: 1) terms describing cancer (“cancer”, “glioblastoma multiforme”, “glioma” and “neoplasm”); 2) terms describing brain tumor (“brain tumor”, “intracranial”); 3) angiogenesis inhibitors (ie, “bevacizumab”, “sorafenib”, “vandetanib”, “pazopanib”, “aflibercept”, “cediranib”, “thalidomide”, “lenalidomide”, “sunitinib”, “axitinib”, “regorafenib”, “ramucirumab”); 5) therapy line (ie, “previously treated”, “refractory”, “second-line”); 6) clinical trials (ie, “prospective”, “clinical”, “human”, or “random”). In addition, we manually searched through abstracts submitted to the 2010 and 2015 American Society of Clinical Oncologists (ASCO) general meeting for applicable trials.
